# Influence of Composite Lay-Up and Cyclic Load Parameters on the Fatigue Behaviour of Flexible Composite Elements

**DOI:** 10.3390/ma17102402

**Published:** 2024-05-16

**Authors:** Lukas Manas, Michal Sedlacik, Martin Ovsik

**Affiliations:** 1 Faculty of Technology, Tomas Bata University in Zlin, Vavreckova 5669, 760 01 Zlin, Czech Republic; lmanas@utb.cz (L.M.); ovsik@utb.cz (M.O.); 2 Centre of Polymer Systems, Tomas Bata University in Zlin, Trida Tomase Bati 5678, 760 01 Zlin, Czech Republic

**Keywords:** flexible element, composite, fatigue behaviour, spring, cyclical stressing

## Abstract

This work is dedicated to the design of flexible composite elements, specifically leaf springs. The design of these flexible composite elements took in consideration the technologies, materials and intermediate goods that are available and useable in laboratory manufacturing and the possibility for the transfer of gained knowledge to industrial practice. This work deals with individual types of materials and their processability and usability for the manufacturing of composite products exposed to cyclic stress. The impact of the designed lay-up diagrams and cyclic load boundary on the fatigue behaviour of manufactured specimens was used to evaluate the effect of cyclic stressing. Based on this assessment, a conclusion and recommendation were formulated for the serial manufacturing of flexible composite elements.

## 1. Introduction

Flexible composite elements, springs, and parabolic flexible elements, for example, leaf and coil composite springs, have been the subject of fundamental and advanced research conducted by research institutions for the better part of the last decade. Activities corresponding to the selection of suitable materials, technology and processes can be observed in some publications concerned with a general introduction to this problematic area. These publications draw their information from several highly specific studies. The core property of flexible elements is their ability to return to their original state after the release of applied force. Current flexible elements allow for the absorption, release or use of both vibrations and impact forces. Among the most elementary properties of springs is their characteristic, which can be progressive, linear or digressive [1,2].

A flexible element can also be defined by the parameter which determines the change of its characteristic dimension in the direction of the acting force, i.e., rigidity. As it is not constant, the rigidity of a flexible object can change during stressing. The inverse value of rigidity is called compliance. The rigidity parameter is dependent on the geometry of the flexible element and its material. The elementary property of a material with regard to its rigidity is its elastic modulus, which describes the relationship between stress and deformation, or elongation per unit length. In general, the elastic modulus of one material is non-changing and depends on varying types of thermal processing. On the opposite side of elastic modulus is the yield strength, which determines the magnitude of stress required for a permanent deformation of the flexible element. Nevertheless, if the induced stress remains below the yield strength, the flexible element will return to its original state once the stress is removed [3,4,5,6].

Another parameter that determines the characteristics of a flexible element is its fatigue limit, which is defined as the stress amplitude that does not lead to fatigue failure. This is constrained by the maximum allowable stress and the initial stress. From a maintenance point of view, the test structure must remain fully useable for a defined number of cycles (greater than 2 × 10^6^), which is dependent on the stress coefficient L_C_, which declines with the increasing number of cycles. The stress coefficient is defined as the ratio of the upper boundary of the loading force F_U_ to the lower boundary of the loading force F_D_, and the value is always higher than 1 [1,7,8,9].

A suitable material satisfying all these requirements can be chosen according to its observed characteristics. From a material aspect, all commonly available reinforcements can be used, which is due to the wide range of properties given by the combinations and compositions of individual layers in composite lay-ups. Among the most used reinforcements are carbon and glass fibres. The early selection of a material must be based on its thermal stability, which is important with regard to its rigidity and other properties. On the other hand, the use of carbon fibres can be limited by their cost, which can favour the use of glass fibres, which have lesser properties in some applications. Jun Ke et al. [10] suggest use of this material, which is capable of improved energy storage in springs and thus has more secure riding capabilities and concurrently causes lesser damage to the roadway. Jun Ke et al. [10] present the advantages of using carbon fibres, although their final recommendation is to use fibres of types E or S2, due to their stated reasons. Furthermore, S2 fibres exhibit superior mechanical properties compared to E fibres; however, E fibres were recommended for use in flexible composite elements as reinforcement due to their lower cost [10,11,12].

It has been demonstrated that the use of a composite flexible element in place of a steel spring can result in a weight saving of at least 50%. This is of particular significance in the aviation and automotive industries, where weight is a crucial factor. Conversely, the producers of cars and railway vehicles have been compelled to reduce the longevity of their products due to cost considerations. The replacement of conventional materials with composite elements would greatly benefit from further studies focusing on the stability of the designed solutions, with a particular emphasis on the safety of the products and their users. Furthermore, composite materials offer greater chemical resistance, electrical resistance, a non-magnetic nature, and natural frequencies [13,14,15].

Flexible composite elements may be a solution for the design of new suspension systems, particularly in the transport industry. One of the earliest instances of the application of flexible composite elements in cars was in 1981, in the Chevrolet Corvette, in which the front and back suspension took advantage of leaf springs. Since then, numerous manufacturers have funded extensive research to use these flexible composite elements in mass production. With consideration of the currently available information, the springs that are currently manufactured are generally dimensioned to deformations corresponding to a loading force of F_MAX_ = 10,000 N. These springs are suitable for use in personal cars or cargo vehicles with lower capacities.

The typical representatives of this group are vans. The innovation investigated in this study is a composite leaf and parabolic spring with progressive characteristics. The European Union established a carbon dioxide target of 95 g/km in 2020. Between 2020 and 2030, it is crucial to reduce the production of CO_2_ by 37.5%. Reaching this goal will continually push manufacturers to innovate and search for new sources of fuel and applications fpr light materials [16,17,18].

The car producer Volvo, in cooperation with SGL Carbon, manufactured one million springs by 17 July 2019. This represented a significant milestone in the mass production of flexible composite components for the automotive industry. This type of spring was constructed with a fabric-reinforced polymer shaped to a preform. This perform was subsequently encased in a matrix in the form of powder and then stabilized by heat in an RTM mould [2,7,18,19].

Sedláček et al. [9] conducted a study investigating the design and optimisation of leaf springs for railway cars. Optimisation was achieved through a numerical simulation based on a conventional solution for steel springs. The new solution was based on epoxy resin and uni-directional glass fibres of type E. The calculations were performed using NX Nasran 11 software. The created FEM model defined the individual layers of the flexible composite structure and was based on the designed geometry of the spring. A 2D model of the spring was created out of CQUAD4 elements. Subsequently, the number of layers and their physical, mechanical and geometrical parameters were defined for various zones within the 2D model [9].

Thippesh [20] experimentally dealt with the replacement of a trapezoid steel spring with a spring made of glass fibres. The spring was composed of uni-directional glass fibres which were saturated with epoxy resin. The discussion in this work focused on the evaluation of the reached stress, frequency and weight of the spring. The supplanted and designed spring were made with a similar geometry and the used materials demonstrated similar mechanical properties. The flexible element was manufactured via a hand lay-up of the dry reinforcement and its subsequent saturation by the matrix. These specimens were then subjected to a standard bending test, which was conducted in accordance with ASTM D790. It was observed that the deformation and maximum stress in the composite leaf springs were consistent with those of steel springs. The overall weight reduction was 80%. The experimental data indicate that the stress and bending of the leaf spring were smaller in comparison to those of steel leaf springs [20].

Similar outcomes were achieved by Heckada [21], who manufactured flexible composite elements from uni-directional glass fibres. The utilisation of these fibres, oriented in one direction, was enabled by the mould, which had a cavity forming a negative imprint of the leaf spring. The designed spring has a potential application as an alternative to conventional steel springs [21].

The submitted work concerns the development and construction solution of composite flexible elements. In particular, this paper describes the fatigue behaviour and influence of lay-up on the number of cycles reached according to pre-determined testing parameters. The design of our flexible elements takes into consideration the technologies, materials and semi-finished goods which are available and useable in serial and laboratory production. This was considered to ensure continuity in case of their possible subsequent application in practice. In the case of composite flexible elements, there must be a distinction between thin-walled and thick-walled structures. The manufacturing cycle of thick-walled elements is more dependent on the optimisation of the lay-up of their individual layers and the optimisation of their curing programme, which must be made with consideration of the structure’s degradation due to the large number of layers.

## 2. Materials and Methods

The materials selected for investigation were chosen according to their mechanical and technological properties, availability and suitability for the mass production of flexible composite structures. Furthermore, the materials were selected based on the findings of previous research.

### 2.1. Material

The practical component of this study employed materials comprising glass fibres as the reinforcement and epoxy resin as the matrix. The first material was provided as a pre-impregnated semi-finished good (prepreg) using plain weave glass fabric (fibre type E-Roving 300 Tex) with the commercial designation TELA (VV320P-DT806R-37) by Delta-Preg (Altopascio, Italy). The second material selected was also a pre-impregnated glass reinforcement (fibre type E-EC9 5x136 Tex/EC9 68 Tex) in a UD variant marked VV430U-DT806R-34. Both products were impregnated with epoxy resin Delta-Tech DT 806R (Sant’Egidio alla Vibrata (TE), Italy). These prepregs have low to medium viscosity and are suitable for curing with vacuum foil in a furnace, autoclave or press, which are all technologies that allow for curing in a matter of minutes. With regard to their processing, these materials are quite universal. The most important material parameters can be seen in Table 1 [22,23].

### 2.2. Design of Flexible Structures

**Figure 1:**
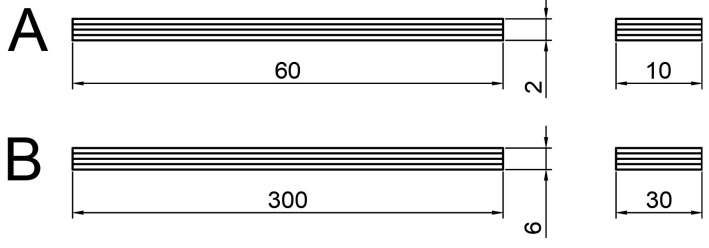
Types of specimens: A—static testing; B—cycling.

For the practical testing part, the flexible composite structures were designed and manufactured into series of specimens, which were used to test their fatigue behaviour. The design of the individual types of specimens that were tested can be seen in Figure 1 in mm. 

Specimen type A was employed to their characterize maximum bending strength, which was quantified in accordance with ČSN EN ISO 14125—fibre-reinforced plastic composites—determination of flexural properties [24].

Specimen type B was employed for laboratory cyclic testing, with the design of the specimens taking into account the equipment available in the laboratories of the Department of Production Engineering (Zlín, Czech Republic). The testing of B-type specimens was conducted on the servo hydraulic testing machine Zwick/Roell HC 25 (Ulm, Germany).

The dimensions of the specimens were selected to align with the selected type of glass reinforcement. The frequency of the prepreg fibres forming the unidirectional fabric was larger in terms of the width of the specimen than if a scaled model of the actual spring had been used. The width of the specimen would be 10 mm if using a scaled-down model of a real spring intended for use on a truck chassis. A single strand of roving approximately 2 mm wide would result in approximately 40% of the fibres of the structure being damaged when cut non-parallel to the strands of the roving. The dimensions of the type B specimens are illustrated in Figure 1.

**Figure 2:**
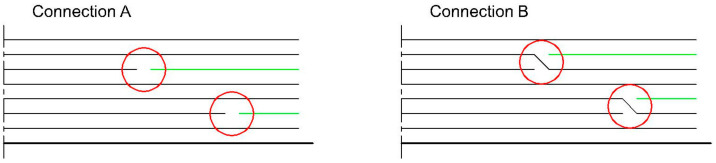
Types of connection: blunt connection (**left**), connection with overlap (**right**).

Three groups of specimens were designed to assess the influence of changes in structure on the fatigue behaviour of type B specimens. The first group contained blunt connections (connection A), while the second group contained connections with overlap (connection B). Finally, the third group of specimens contained no connection, only full plies. Figure 2 illustrates the connections A and B.

**Figure 3:**
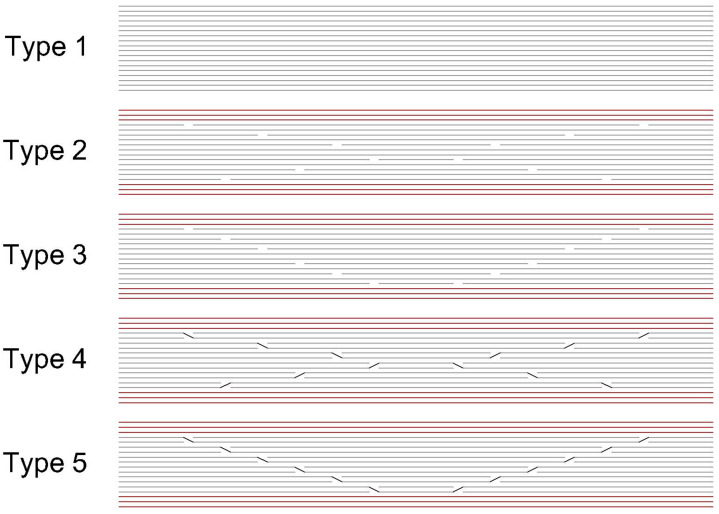
Lay-up diagrams—structures with no connection (Type 1); placement in X shape (Type 2, Type 4) and pyramid outline (Type 3, Type 5).

Five types of structures were prepared to evaluate the fatigue behaviour of the material. Their corresponding lay-up diagrams are presented in Figure 3. The designs had the connections arranged in either an X shape or pyramid shape. The placement of these connections was carried out according to the available research papers and empirical experience that corresponds with the generally accepted methods used in the design of the ply lay-ups in composite structures. The structure with no connections was selected as the reference sample. This structure was designated as Type 1. The structure comprised 18 layers of the selected material.

The evaluation of fatigue behaviour was performed on following structure types:

- Type 1—Structures with no connections;
- Type 2—Blunt connections—X grouping;
- Type 3—Blunt connections—pyramid grouping;
- Type 4—Connections with overlap—X grouping;
- Type 5—Connections with overlap—pyramid grouping.

**Figure A1:**
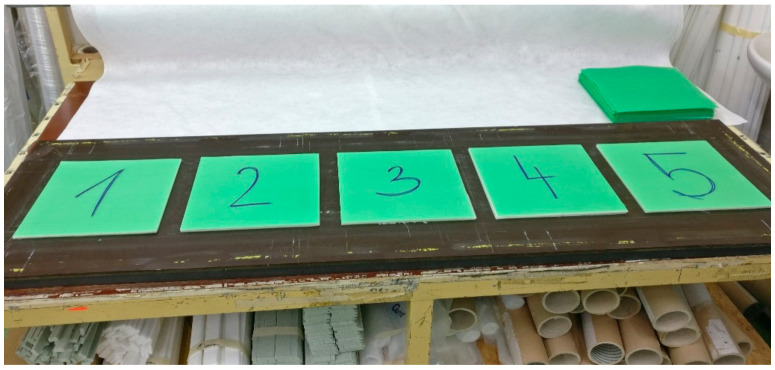
Laying of layers for structures of Type 1–5.

**Figure A2:**
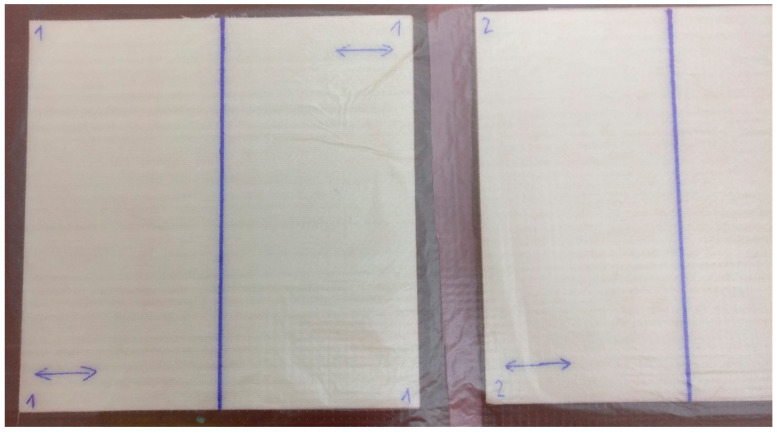
Detail of lay-up of flexible composite element.

The individual layers of the presented structures (types 2-5) were laid in accordance with the laying schemes detailed in the Appendix A. The structures containing blunt connections can be seen in Table A1 and Table A2. The structures containing connections with overlap can be seen in Table A3 and Table A4. Further details on both lay-up methods can be found in the manual provided in the Appendix A. Figure A1 illustrates the application of prepreg material to the mould, while Figure A2 provides a detailed illustration of the orientation of the fibres and the centre of the structure, which facilitates an understanding of the process.

### 2.3. Preparation of Flexible Structures

The designed specimens and flexible structures were sealed in a vacuum bag and manufactured in a curing furnace with a constant negative pressure (0.85 MPa). This manufacturing technology was selected due to its suitability for laboratory conditions and the number of manufactured structures made.

A flat mould was prepared from Kartit material for the manufacture of the flexible composite elements. Kartit is composed of cellulose paper (reinforcement) and formaldehyde resin, which acts as a binder. Plates of this material are frequently employed in the processing of composite materials and can be suitable for flat products if a suitable separator is selected. One of its advantages is that it has a low weight in comparison to steel. The rigidity of an assembly comprising this plate and a simple steel frame is sufficient to allow for straightforward manipulation, even when working with large products.

The selected mould was initially cleaned with isopropyl alcohol, after which a penetration layer (Loctite Frekote B 15 (Düsseldorf, Germany)) was applied to fill the pores in the mould. Two further penetration layers were applied after 30 min. The treated mould was then left to cure at ambient temperature for 24 h. Subsequently, a semi-permanent separator (Loctite Frekote 700-NC (Düsseldorf, Germany)) was applied in four layers over a five-minute interval. The manufacturer has set the curing time for the final layer at 20 min at ambient temperature.

Following the preparation of the mould, the lay-up of the composite materials commenced. In the case of prepreg materials stored at −18 °C, the manufacturer recommends tempering the prepreg for a minimum of six hours before use. After the 6 h period, the work commenced with the removal of the protective polyethylene foil which prevents the non-cured material from absorbing aerial humidity during tempering, thus improving the quality of manufactured parts. The next step included cutting the prepreg, which was achieved with the Zünd M-1600 CV plotter (Altstätten, Switzerland), in accordance with the cutting plan. The prepreg was cut in a manner that permitted the creation of specimens with the specified dimensions (310 × 310 × 6) mm.

The cut material was divided according to the designed lay-up diagrams for individual specimens. The divided composite materials were subsequently laid on the manufacturing mould that had been prepared beforehand. The first layer was placed in the designated position, which allowed for the easier use of the auxiliary materials needed for the proper sealing of the composite product. The remaining plies were laid subsequently.

Prepared layers of material were covered by micro porous polypropylene flexible foil PP40, which is used to create an interface between the release fabric and the prepreg. The release fabric used in this work (areal weight 83 g/m^2^ and thermal stability up to 200 °C) was made from Nylon 66. The use of micro porous foil is recommended especially for thin-walled or non-flat parts which could be damaged during the removal of auxiliary material. The final auxiliary materials used in this lay-up were a bleeder and vacuum foil.

**Figure 4:**
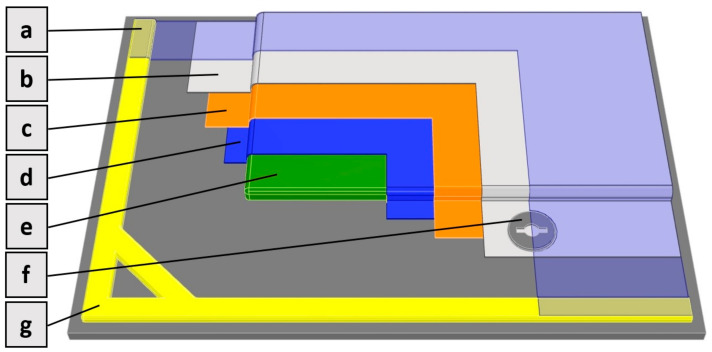
Scheme of lay-up: (a) vacuum foil; (b) bleeder; (c) peel ply; (d) release film; (e) prepreg material; (f) valve; (g) sealing tape.

The bleeder 150 UVLS 200, at 150 g/m^2^, was used in the preparation of the specimens. The flexible polyamide vacuum foil PA180 was utilised as the cover layer of the prepreg plies. This is a universal multi-layer foil with thermal stability up to 180 °C. The sealing element employed in this study was heat-resistant butyl rubber tape, and the requisite quality of the lay-up was ensured by a 30 mm bridge in between the sealing element and vacuum bag. The visualization of this bridge can be seen in Figure 4, specifically in the space between the sealing element (g) and bleeder (b).

The process of vacuuming is enabled by two-part valves saddled with a universal coupler for air systems. For the vacuuming of larger parts, a larger number of valves is required for the more effective outlet of air, or even for the compensation of small leaks in sealing system. The valves should be equipped with a manometer to properly measure the negative pressure.

Upon completion of the lay-up, it was placed into a tempered furnace. The specimens were manufactured in a heated furnace with a controlled heating cycle. This function enables the selection of an appropriate ramp up to the curing temperature, hold on the curing temperature and subsequent controlled cooling to the de-mould temperature. The curing program was chosen according to the material sheet, which was supplied by the prepreg manufacturer.

The curing program used to manufacture type A and B contained following steps:

- Heat material by 2 °C/min from laboratory temperature to 65 °C;
- Hold at 65 °C for 30 min;
- Heat material by 2 °C/min to curing temperature (100 °C);
- Hold at 100 °C per 90 min;
- Cool down by 2 °C/min to laboratory temperature.

In order to achieve the maximum Tg, the manufacturer recommends the addition of post-curing. This process involves the continuous heating of the part to the curing temperature, which is then followed by additional heating at a slow ramp up (generally 0.5 °C/min). This method can be employed to enhance the maximum glass transition temperature of the manufactured part, thus preventing potential deformations or internal stresses.

The parameters for post-curing are as follows:

- Heat part by 2 °C/min to curing temperature (100 °C);
- Heat part by 0.3 °C/min to 120 °C;
- Hold at 120 °C for 60 min;
- Cool down by 2 °C/min to laboratory temperature.

**Figure 5:**
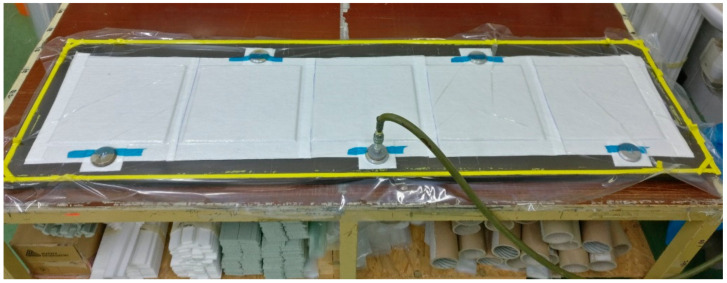
Vacuumed material.

Figure 5 depicts an image of the vacuumed structures prepared on a flat mould prior to their insertion into the tempered furnace.

**Figure 6:**
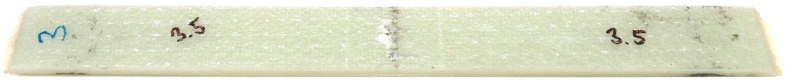
Flexible composite specimen.

Following the curing process, the manufactured plates were de-moulded and prepared for further processing. Any residual auxiliary materials were segregated and disposed of in the appropriate waste bins for recycling. A prepared composite plate can be seen in Figure 6.

### 2.4. Cyclic Fatigue Test

The fatigue test was performed under laboratory conditions on the dynamic servo hydraulic testing machine Zwick/Roell HC 25 (Ulm, Germany). The main requirement was to achieve the desired rigidity of the flexible element (required value 150 N/mm) and its longevity (200,000 cycles) at its upper load boundary and our chosen frequency. For the results of conventionally used flexible elements to be comparable to those of the proposed composite elements, it is necessary to achieve the required number of cycles at a stress approaching 500 MPa. This value corresponds to a load level of 70% (514 MPa). The value of the cycling frequency was chosen to be 1.5 Hz (requirement: 0.5–2.5 Hz). These are the general requirements defined by the manufacturer of the flexible elements used in truck chassis. The chosen frequency of testing should be as high as possible unless the results of the experiment may be affected. Fewer cycles and a lower frequency should usually be chosen for a wider range of stress. On the other hand, more cycles and a higher frequency should be chosen for a narrower range of stress. A higher frequency of testing can lead to more cycles, and thus increase longevity [25].

In addition, inner frictional energy induced by the mutual movement of delaminating layers can be created. At lower scales, this energy is created by friction between the reinforcement and matrix. The tested structures can also be affected by viscoelastic hysteresis. Heating by friction and viscoelastic hysteresis can be hard to quantify during temperature measurements. Dynamic testing has not proved whether there is a significant thermal effect on the structures at the observed points affected by frictional forces. If a shorter fatigue longevity is assumed, then the suitable frequency range is 1–2 Hz. On the contrary, the suitable frequency range for more cycles is 3–8 Hz [25].

The varied parameters in the fatigue testing of type B specimens inputted to the TestXpert R (V1.82-EN) testing machine (Ulm, Germany) were frequency and load level. The control program was configured in the Sequence editor dialog box.

The programme was divided into eight phases. Phases 1 to 5 were designated for the setting of the machine before the beginning of testing, the zeroing of observed values and setting of the conditions for the premature termination of testing; for example, if a specimen of a different geometry was placed into the testing rig. Phase 6 of the testing program served to set up the conditions of the testing program and its individual limitations. The set up was important especially for frequency, lower and upper boundary of the load and the maximum number of cycles. Another important step was the determination of the conditions and actions executed if the limitations were breached. The breaching of individual limitations led to phase 7, or possibly phase 8—the termination of the testing program. The testing programme controls the load F [N] given by the amplitude of the load for the mean value of loading, which is designated by the software TestXpert R (V1.82-EN) as the “Mean value Force” [N].

The values of F_MAX_ [N] for the specimens were determined based on statistic testing. Varying values of the load coefficient were also used during testing.

The test parameters were inputted to the Sequence editor in the TestXpert R program before the experiment commenced. These values can be seen in Table 2, Table 3, Table 4 and Table 5. The testing was divided into three parts. The first part assessed the effect of the type of connection on the achieved number of cycles. The second and third parts assessed the effect of changing the cyclic load parameters on the fatigue behaviour of the flexible composite structures. The parameters are related to the characteristics of the vehicle set. The change in load on the axle elements of the vehicle due to the influence of the upper and lower load boundary levels is a real phenomenon that occurs when the vehicle is in motion. These phenomena occur due to the loading and unloading of the system.

Table 2 illustrates the parameters for the fatigue behaviour testing included in the first phase of the testing. This configuration was implemented for specimens with a connection within their inner structure. The demonstrated configuration was also utilised for the group of specimens designated as Type 1. These are the parameters for Type 1–5. A total of five specimens were tested for each type.

**Table 2:** Parameters of the first phase testing for Type 1–5 structures.

	Designation	Parameter
Maximum force	F_MAX_ [kN]	2.5
Loading level	[%]	70
Supports spacing	l_s_ [mm]	250
Frequency	[Hz]	1.5
Upper load boundary	F_U_ [kN]	1.68
Lower load boundary	F_D_ [kN]	0.05
Load coefficient	L_C_	33.6
Number of samples	pcs	5

The settings of the parameters for the second phase of the testing are presented in Table 3. This table displays the parameters associated with the fatigue behaviour of structures with no connections in their inner structure (Type 1). One of the parameters that underwent variation during the second part of the experiment focused on the description of structures without connections was the load level. In total, six load parameters were altered in order to determine the structures’ S-N curves.

The alteration of this parameter was found to be proportionate to changes in the parameters of the upper load boundary and load coefficient. For each change in the loading level parameter, five samples were tested.

**Table 3:** Load level (upper load boundary) parameters for the testing of Type 1 structures.

	Designation	Parameter	Parameter	Parameter
Maximum force	F_MAX_ [kN]	5		
Load level	[%]	100	90	80
Supports’ spacing	l_s_ [mm]	125		
Frequency	[Hz]	5		
Upper load boundary	F_U_ [kN]	5.0	4.5	4.0
Lower load boundary	F_D_ [kN]	1		
Load coefficient	L_C_	5.0	4.5	4.0
Number of samples	pcs	5	5	5
	Designation	Parameter	Parameter	Parameter
Maximum force	F_MAX_ [kN]	5		
Load level	[%]	70	60	50
Supports’ spacing	l_s_ [mm]	125		
Frequency	[Hz]	5		
Upper load boundary	F_U_ [kN]	3.5	3.0	2.5
Lower load boundary	F_D_ [kN]	1		
Load coefficient	L_C_	3.5	3.0	2.5
Number of samples	pcs	5	3	0

Table 4 and Table 5 present the parameters for the fatigue testing conducted in the third phase of the study, which was designed to assess the behaviour of the Type 1 structure under specific loading conditions. In this phase of testing, the lower load boundary was adjusted to the selected load levels of 60% and 70%. The change in the lower load boundary was made in a proportionate manner, corresponding to the change in the load coefficient. It should be noted that the load coefficients were intentionally identical for both selected loading levels. In the third phase of testing, three specimens were evaluated for each change in the lower load boundary.

**Table 4:** Lower load boundary parameters for the testing of Type 1 structures—upper load boundary, 3 kN.

	Designation	Parameter	Parameter	Parameter
Maximum force	F_MAX_ [kN]	5		
Load level	[%]	60		
Supports’ spacing	l_s_ [mm]	125		
Frequency	[Hz]	5		
Upper load boundary	F_U_ [kN]	3		
Lower load boundary	F_D_ [kN]	0.429	0.214	0.043
Load coefficient	L_C_	7	14	70
Number of samples	pcs	3	3	3

**Table 5:** Lower load boundary parameters for the testing of Type 1 structures—upper load boundary, 3.5 kN.

	Designation	Parameter	Parameter	Parameter
Maximum force	F_MAX_ [kN]	5		
Load level	[%]	70		
Supports’ spacing	l_s_ [mm]	125		
Frequency	[Hz]	5		
Upper load boundary	F_U_ [kN]	3.5		
Lower load boundary	F_D_ [kN]	0.500	0.250	0.050
Load coefficient	L_C_	7	14	70
Number of samples	pcs	3	3	3

**Figure 7:**
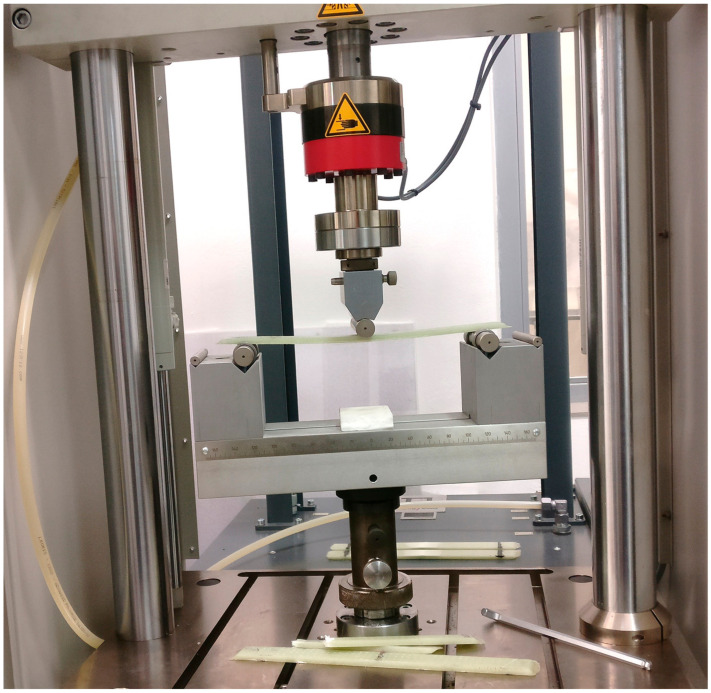
Servo-hydraulic testing device Zwick/Roell HC 25.

**Figure 8:**
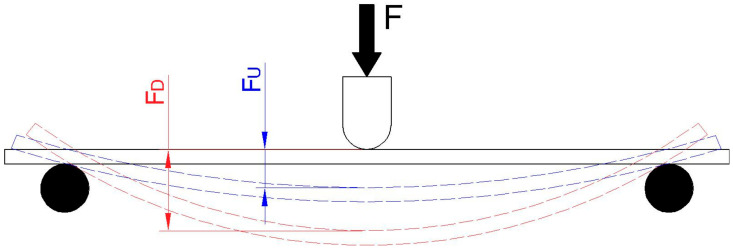
Schematic of cycling.

Once the parameters had been defined, the bending test apparatus was positioned between the main plate and the beam. Figure 7 depicts the testing equipment. Figure 8 also illustrates the upper load boundary F_D_, lower load boundary F_U_ and the load F.

## 3. Results

*Cyclic Fatigue Test Results*

This chapter presents the results for structures tested with the different settings provided in the tables. The parameters which were varied in different settings, and mean values, are bolded. Among the main results is the fact that the number of cycles (repetitions) corresponds to changes made in load levels.

Table 6 illustrates the number of repetitions achieved in the context of reference structure Type 1. The reached repetitions in the testing of the fatigue behaviour of structures containing connections within their inner structure can be seen in Table 7 and Table 8.

Table 9 illustrates the number of cycles (repetitions) achieved for structures with varying upper load boundaries, corresponding to the changes in their load levels. For a load level of 60%, three samples were tested due to the number of cycles they achieved. Their achieved values are well above the required parameter.

Table 10 and Table 11 illustrate the number of cycles achieved for structures with varying lower load boundaries, measured at a fixed load level.

The results of the fatigue testing can be divided into two groups. The first group contains the results of structures with connections within their inner structure, while the second group contains structures without connections. As is evident from the achieved average value of repetitions, specimens containing connections within their inner structure demonstrated a lower number of reached cycles. For comparison, the reference structure exposed to the upper load boundary (521 MPa) held for almost 70 thousand cycles. Conversely, structures containing blunt connections (types 2 and 3) and connections with overlap (Type 4) demonstrated a 25% reduction in their number of cycles.

The exception to this was the structure containing connections with overlaps grouped into a pyramid (Type 5), which demonstrated 5% fewer cycles reached in comparison to the reference structure (Type 1). This was caused by the lay-up diagram of structure Type 5 and the connection, with overlap, itself. The specimens with this type of connection reached 19% more cycles than the specimens with blunt connections with a pyramid distribution. Conversely, specimens comprising connections with overlap grouped into a pyramid reached 5% more cycles than those with their distribution in an X shape.

The results indicate that specimens containing connections with overlap exhibited higher toughness, particularly in comparison to specimens containing blunt connections. Furthermore, the structures of Type 5 containing connections with overlap demonstrated a lower variation coefficient or dispersion of values. This result corroborates the hypothesis that optimising a structure can lead to a higher number of cycles, and thus increased longevity. The second group of values contains results for the part of the measurement that considers the change of load level and lower load boundary, which is also connected with the change of load amplitude. The second group is made of samples that are half the size of the first group, although the results of their individual load levels can be grouped together. At a load level of 70%, the tension in the structure for specimens of half size was 514 MPa. A comparison of the results obtained for specimens tested at the same load level reveals a significant degree of variation. This variability was a consequence of the setting of the load coefficient L_C_ (33.6 for the reference specimen), which was selected to reduce the testing time required for the characterisation of all specimens from the prepared series.

**Figure 9:**
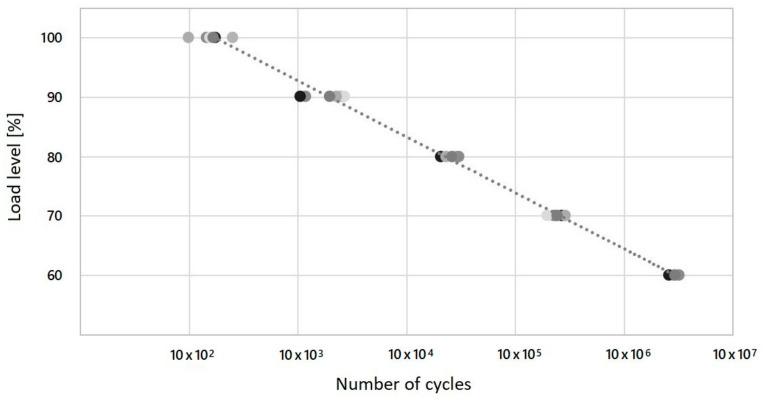
S-N curve of compared structures—number of cycles at different load levels.

The number of cycles shown in Table 9 describes the transition from low-cyclic behaviour to high-cyclic behaviour of Type 1 samples. At a load level of 100% of the maximum force determined by the static test, the results were in the range of hundreds of cycles. In contrast, at a 60% load level, the number of cycles was in the range of millions. This is a characteristic of and desired course for individual load levels. The behaviour of these structures, namely the dispersion of their values at individual load levels, can be observed in their S-N diagram (Figure 9). The diagram displays the S parameter, which signifies the cyclic load, and the N parameter, which signifies the repetition of the defined cyclical load. The achieved number of cycles for each load level (five cycles for each load level) is displayed in shades of grey in order to enhance the readability of the data presented in the figure.

The S-N curve and the extrapolation, or definition, of the model’s development generally divides the axis of the longevity of the evaluated specimen into three sectors. These sectors are further divided into low, medium, and high values of the number of cycles reached. The medium value is between the high and low number of cycles. The described trend is also apparent in the results. As shown in Figure 9, the results of the structures are more dispersed at higher load levels than at lower ones. The reason for this is that the final fatigue of the structures under compression is defined as a combination of fatigue and the buckling induced by delamination. The dispersion of values is caused by numerous damage mechanisms and a lower measurement volume, which is costly from both an economic and a time perspective. The values of the variation coefficients can be seen in Table 9.

**Figure 10:**
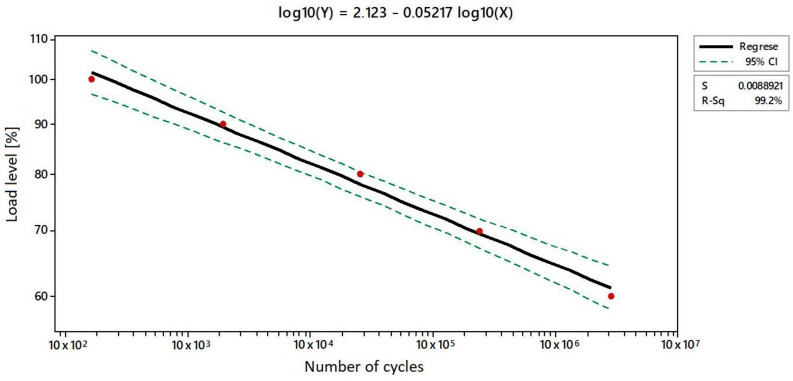
Determination of load level interval [%] for investigated number of cycles.

The objective of S-N curves is to characterise and predict the average fatigue longevity of cyclic loads. The fatigue behaviour of their material is therefore crucial for the design of flexible composite elements. However, it should be noted than a certain percentage of imperfections should be expected in the results. Consequently, the degree of reliability for individual load levels was defined. Figure 10 shows the Wöhler curve plotted on a logarithmic scale, from which the interval for the load levels was determined. The value of the reliability of the average number of cycles reached for individual load levels was R^2^ = 0.992. R^2^ is significantly close to 1, indicating a greater than 99% correlation with the observed properties. The median of the values was not used for the creation of this curve, as the values of the average and median in Table 9 are quite close.

In order to ascertain the requisite value of the load level interval, the following equation was employed:

log (Y) = 2.123 − 0.05217 log (X)(1)

in which Y is the load level [%] and X is the number of cycles. There is a 95% probability that the values of the number of cycles fit within the plotted confidence intervals projected on the axis representing the load level.

**Figure 11:**
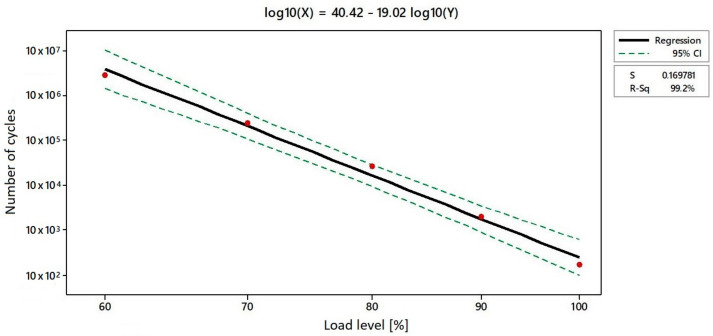
Determination of number of cycles interval for investigated load levels [%].

Figure 11 illustrates the logarithmic regression curve that was employed to ascertain the requisite values of the interval for the number of cycles. The mathematical model is defined by the following logarithmic equation:

log (X) = 40.42 − 19.02 log (Y)(2)

in which X represents the reached number of cycles, while Y represents the load level [%]. Once again, there is a 95% probability that values of the number of cycles fit within the plotted confidence intervals projected on the axis representing the numbers of cycles.

As a matter of fact, S-N diagrams are generally plotted for corresponding load coefficients, while, here, only the S-N diagram for specimens with varied load levels was evaluated (Table 9).

The alteration in the lower load limit for specimens with no inner connections (Type 1) exemplifies the influence of load factor on the resulting number of cycles. The selection of a lower limit value of 50 N is rational, given that this is a model case occurring within the duty cycle of a rig utilising a flexible element.There can be a situation during motion in which the entire riding system is relieved of stress almost completely, for example, due to an uneven riding surface. In this scenario, the lower load boundary approaches zero. The results of the fatigue behaviour testing indicate a potential decrease of up to 9% in the number of cycles reached when the lower load boundary declines from 500 N to 250 N, and up to 5% for a change from 250 N to 50 N for a 70% load level. Furthermore, a reduction from 1000 N to 50 N resulted in a decline of 45%.

In the case of structures tested at a load level of 60%, a change in the lower load boundary from 1000 N to 50 N resulted in a decrease in longevity of 85%, i.e., from 2,893,012 cycles to 448,850. Therefore, it can be concluded that a change in the lower load boundary directly affects the longevity of structures. Higher numbers of cycles were reached by structures with no connections within their inner structure. Nevertheless, in the design of composite flexible elements, it is not assumed that this value of the lower load boundary will be achieved throughout the life cycle of the vehicle.

**Figure 12:**
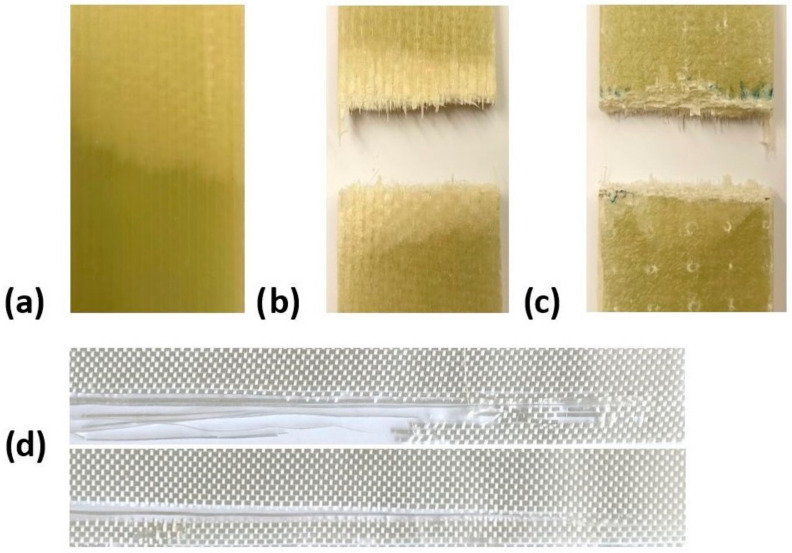
Types of failures seen after testing: (**a**) delamination; (**b**) tension side; (**c**) compression side; (**d**) damage to layers.

Following the experiment, the defects of the tested specimens were evaluated. Figure 12 illustrates the visible defects on the composite specimen, with its compression (c) and tension sides (b) clearly visible. At the point of failure, compression (compression side) and fibre tearing (tension side) occur. The internal failure caused by the delamination of the material layers in the structures with blunt connections is illustrated in part a (Figure 12). To assess the damage to the fabric of the individual layers of the flexible structure, the epoxy resin was removed in an oven by heat. The images displayed in Figure 12 display a part of the flexible composite element.

**Figure 13:**
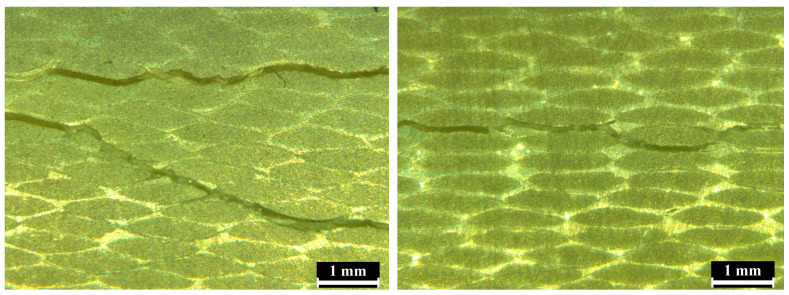
Internal failures after testing.

The evaluation of the individual layers of the glass fabric led to the use of microscopic imaging. Samples were taken from sections of the test specimens at the point of failure and ground and polished. They were then investigated using a Leica DMI 3000 M microscope with a digital camera. The defects are shown in Figure 13. Regarding thick-walled flexible composite structures, it can be concluded that the placement of individual layers can influence the resulting thickness of the structure. This phenomenon can occur when the individual layers are not precisely aligned, with the rovings positioned on top of each other in a uniform manner.

## 4. Conclusions and Discussion

The fatigue behaviour of flexible composite structures was observed in several types of specimens. The testing was conducted using laboratory tools at the Department of Production Engineering (Zlín, Czech Republic). The parameters of individual tests were selected with regard to static test results and the requirements demanded of flexible composite elements and conventional flexible elements by their producers.

The results of the fatigue behaviour testing demonstrate possibilities for the use of the designed structures at the 70% load level (514 MPa). At this load level, with the consideration of possible changes to the lower load boundary, it is possible to achieve the determined number of cycles (200,000).

As shown in the results of type B structures, the incorporation of optimisation into thick-walled structures containing connections with overlap positively influenced the value of the reached number of cycles. Furthermore, it can be expected that the reached number of cycles increases with use of lower load levels. The manufacturing of thick-walled structures also strongly benefits from the optimisation of the curing program. In general, this operation is performed by a gradual lay-up of prepreg layers on a prepared mould with the frequent use of semi-vacuation. This method enables the elimination of any possible defects in the structure and, at the same time, the prevention of cohesion loss in between the individual layers during the testing of the flexible composite structures.

The measured results indicate that use of structures manufactured exclusively from uni-directional fibres can positively influence the reached number of cycles.

This claim was confirmed by an evaluation of the defects created on the outside and the inside of the structure. The use of uni-directional reinforcement led to the improved interconnectivity of individual prepreg layers. The results from the first part of the experiment demonstrated how the lay-up of individual layers influences the overall cohesion of the structure. There may exist some critical points which can generate inner tension that might lead to a delamination of the layers and subsequent complete loss of stability of the flexible element. It is better to use structures with no connections or connections with an overlap of longer and shorter prepreg cuts to create a parabolic structure.

This can be achieved by a change in the geometry of the cross-section in the longitudinal direction, so that the spring is parabolically shaped and contains same volume of fibres all along its length. In conclusion, the structure changes the profile width with increasing distance from the centre of the spring.

For this application, it was necessary to create a cavity mould with a geometry corresponding to the negative of the intended shape of the flexible composite element.

The change of the upper and lower boundaries of loading shows the influence of these parameters on the reached number of cycles. The changing of the upper parameter—load level—led to the determination of the longevity curve. It was confirmed that the change in the reached number of cycles is dependent not only on the acting load induced by a moving vehicle, but also on the lower load boundary, which is induced by the full weight of the vehicle or the release of this weight during motion. The logarithmic expression of this event can help with the determination of confidence intervals for the investigated number of cycles, and eventually the investigated load level.

The other option for increasing the load capacity is to use glass reinforcement type S2, which in general reaches a higher strength than glass reinforcement type E. For this purpose, it would be necessary to design a suitable prepreg with this glass reinforcement and select a suitable supplier.

As evidenced by sources from the literature and exemplified in the real-world applications of flexible elements, it is feasible to utilise structures with an internal pre-stress that exhibits a progressive characteristic.

The future direction of this problematic area for flexible composite structures could focus on the design of thick-walled structures and the verification of their manufacturing, with the application of the knowledge gained in this research.

## Figures and Tables

**Table 1:** Material properties.

Reinforcement	Fabric Warp	Areal Weight of Reinforcement [g/m^2^]	Weight Ratio in x and y Axes [%]	Fibre Type
VV320P	Plain TELA	320	50/50	Roving 300 tex
VV430U		425	90/10	EC95x136 tex EC9 68 tex
Reinforcement	Resin type	Curing temperature [°C]	Curing time [hour]	Resin content [%]
VV320P	DT 806R	65–140	0.1–16	37
VV430U				34

**Table 6:** Number of cycles—repetitions—achieved in Type 1 structure with no connection.

Structure Type	Repetitions
Type 1	66,969
	59,957
	69,511
	72,411
	80,336
Average	69,837
Variation coefficient [%]	9.56
Minimum	59,957
Median	69,674
Maximum	80,336

**Table 7:** Number of cycles—repetitions—achieved with blunt connections: Type 2 and Type 3.

Structure Type	Repetitions	Structure Type	Repetitions
Type 2	58,703	Type 3	52,221
	53,558		57,373
	36,802		53,644
	63,597		61,886
	40,767		45,151
Average	50,685	Average	54,055
Variation coefficient [%]	20.32	Variation coefficient [%]	10.31
Minimum	36,802	Minimum	45,151
Median	52,122	Median	53,850
Maximum	63,597	Maximum	61,886

**Table 8:** Number of cycles—repetitions—achieved with connections with overlap: Type 4 and Type 5.

Structure Type	Repetitions	Structure Type	Repetitions
Type 4	53,360	Type 5	73,755
	46,134		64,632
	58,645		eliminated
	63,715		63,256
	44,965		64,916
Average	53,364	Average	66,640
Variation coefficient [%]	13.46	Variation coefficient [%]	6.24
Minimum	44,965	Minimum	63,256
Median	53,362	Median	64,916
Maximum	63,715	Maximum	73,755

**Table 9:** Number of cycles—repetitions—achieved by Type 1 structures with a varying upper load boundary.

Upper load boundary F [kN]	5.0	4.5	4.0	3.5	3.0
Lower load boundary F [kN]	1.0				
Load level [%]	100	90	80	70	60
Specimen 1	250	2525	28,597	236,879	2,838,846
Specimen 2	146	1202	30,195	225,313	3,250,874
Specimen 3	175	1054	20,801	268,405	2,589,317
Specimen 4	156	2701	26,360	197,251	-
Specimen 5	98	2259	23,418	286,343	-
Average [Repetitions]	165	1948	25,874	242,838	2,893,012
Variation coefficient [%]	33.55	39.36	14.73	14.51	11.55
Minimum [Repetitions]	98	1054	20,801	197,251	2,589,317
Median [Repetitions]	156	2259	26,260	236,879	2,838,846
Maximum [Repetitions]	250	2701	30,195	286,343	3,250,874

**Table 10:** Number of cycles—repetitions—achieved by Type 1 structure. Load level 70% and varying lower load boundary.

Upper load boundary F [kN]	3.5		
Lower load boundary F [kN]	0.500	0.250	0.050
Load level [%]	70		
Specimen 1	163,071	154,083	128,522
Specimen 2	123,268	137,992	131,007
Specimen 3	170,358	126,455	139,776
Average [Repetitions]	152,232	139,510	133,102
Variation coefficient [%]	16.65	9.95	4.44
Minimum [Repetitions]	123,268	126,455	128,522
Median [Repetitions]	163,071	137,992	131,007
Maximum [Repetitions]	170,358	154,083	128,522

**Table 11:** Number of cycles—repetitions—achieved by Type 1 structure. Load level 60% and varying lower load boundary.

Upper load boundary F [kN]	3		
Lower load boundary F [kN]	0.500	0.250	0.050
Load level [%]	60		
Specimen 1	679,881	594,813	550,822
Specimen 2	655,770	531,977	402,804
Specimen 3	866,998	520,783	392,925
Average [Repetitions]	734,216	549,191	448,850
Variation coefficient [%]	15.75	7.27	19.71
Minimum [Repetitions]	655,770	520,783	392,925
Median [Repetitions]	679,881	531,977	402,804
Maximum [Repetitions]	866,998	594,813	550,822

## Data Availability

Data are contained within the article.

## Appendix A

Table A1 shows the lay-up system for the laying of prepreg material for Type 2 specimens (blunt connection, grouping in X shape). The principle of this method lies in keeping the order of the following lay-up:

- Laying of 1st layer—length 300 mm;
- Laying of 2nd layer—length 300 mm;
- Laying of 3rd layer—length 300 mm;
- Laying of 4th layer, composed of three segments creating two blunt connections—the length of layers was 37.5 mm on the right side, 225 mm in the middle and 37.5 mm on the left side;
- Laying of 5th layer—length 300 mm;
- Laying of final layer.

**Table A1:** Order of lay-up for Type 2 structures.

Type 2—Blunt Connection, Grouping in X Shape				
Layer	Left Side [mm]	Centre [mm]	Right Side [mm]	Layer Length [mm]
1.	-	300	-	300
2.	-	300	-	300
3.	-	300	-	300
4.	37.5	225	37.5	300
5.	-	300	-	300
6.	75	150	75	300
7.	-	300	-	300
8.	112.5	75	112.5	300
9.	-	300	-	300
10.	-	300	-	300
11.	131.25	37.5	131.25	300
12.	-	300	-	300
13.	93.75	112.5	93.75	300
14.	-	300	-	300
15.	56.25	187.5	56.25	300
16.	-	300	-	300
17.	-	300	-	300
18.	-	300	-	300

The same method was used for the Type 3 lay-up (blunt connection with pyramid grouping), which is shown in Table A2.

**Table A2:** Order of lay-up for Type 3 structures.

Type 3—Blunt Connection, Pyramid Grouping				
Layer	Left Side [mm]	Centre [mm]	Right Side [mm]	Layer Length [mm]
1.	-	300	-	300
2.	-	300	-	300
3.	-	300	-	300
4.	37.5	225	37.5	300
5.	-	300	-	300
6.	56.25	187.5	56.25	300
7.	-	300	-	300
8.	75	150	75	300
9.	-	300	-	300
10.	-	300	-	300
11.	93.75	112.5	93.75	300
12.	-	300	-	300
13.	112.5	75	112.5	300
14.	-	300	-	300
15.	131.25	37.5	131.25	300
16.	-	300	-	300
17.	-	300	-	300
18.	-	300	-	300

Table A3 and Table A4 show the lay-up system for the laying of prepreg material for Type 4 (connections with overlap, grouping in X shape) and Type 5 specimens (connections with overlap, grouping in pyramid shape). The principle of this method (Table A3) lies in keeping the order of the following lay-up:

- Laying of 1st layer—length 300 mm;
- Laying of 2nd layer—length 300 mm;
- Laying of 3rd layer—length 300 mm;
- Laying of 4th layer—length 225 mm;
- Laying of 5th layer—length 300 mm;
- Laying of 6th layer—length for both left and right side 37.5 mm;
- Laying of 7th layer—length 150 mm;
- Laying of 8th layer—length 300 mm;
- Laying of final layer.

**Table A3:** Order of lay-up for Type 4 structures.

Type 4—Connections with Overlap, Grouping in X Shape				
Layer	Left Side [mm]	Centre [mm]	Right Side [mm]	Layer Length [mm]
1.	-	300	-	300
2.	-	300	-	300
3.	-	300	-	300
4.	-	225	-	225
5.	-	300	-	300
6.	37.5	-	37.5	75
7.	-	150	-	150
8.	-	300	-	300
9.	75	-	75	150
10.	-	75	-	75
11.	-	300	-	300
12.	112.5	-	112.5	225
13.	131.25	-	131.25	262.5
14.	-	300	-	300
15.	-	37.5	-	37.5
16.	93.75	-	93.75	187.5
17.	-	300	-	300
18.	-	112.5	-	112.5
19.	56.25	-	56.25	112.5
20.	-	300	-	300
21.	-	187.5	-	187.5
22.	-	300	-	300
23.	-	300	-	300
24.	-	300	-	300

**Table A4:** Order of lay-up for Type 5 structures.

Type 5—Connections with Overlap, Grouping in Pyramid Shape				
Layer	Left Side [mm]	Centre [mm]	Right Side [mm]	Layer Length [mm]
1.	-	300	-	300
2.	-	300	-	300
3.	-	300	-	300
4.	-	225	-	225
5.	-	300	-	300
6.	37.5	-	37.5	75
7.	-	187.5	-	187.5
8.	-	300	-	300
9.	56.25	-	56.25	112.5
10.	-	150	-	150
11.	-	300	-	300
12.	75	-	75	150
13.	-	112.5	-	112.5
14.	-	300	-	300
15.	93.75	-	93.75	187.5
16.	-	75	-	75
17.	-	300	-	300
18.	112.5	-	112.5	225
19.	-	37.5	-	37.5
20.	-	300	-	300
21.	131.25	-	131.25	262.5
22.	-	300	-	300
23.	-	300	-	300
24.	-	300	-	300
